# A Rare Breast Tumor Presenting As Adenomyoepithelioma With Suspicious Imaging Features: A Case Report

**DOI:** 10.7759/cureus.82450

**Published:** 2025-04-17

**Authors:** Jose C Ramos, Anjeza Chukus

**Affiliations:** 1 Diagnostic Radiology, Aventura Hospital and Medical Center, Aventura, USA; 2 Neuroradiology, Aventura Hospital and Medical Center, Aventura, USA

**Keywords:** biphasic tumor, bi-rads 4, breast adenomyoepithelioma, epithelial-myoepithelial lesion, histopathology, intraductal papilloma, ultrasound-guided core biopsy

## Abstract

Adenomyoepitheliomas (AMEs) are rare breast neoplasms often detected incidentally during routine screening mammograms in asymptomatic females. Clinically, AMEs usually present as a palpable, solitary mass, though they may also be discovered during routine imaging. Radiologically, AMEs exhibit variable features. Mammography findings may include round or lobulated masses with circumscribed or indistinct margins, occasionally accompanied by microcalcifications. On ultrasound, they typically appear as solid, hypoechoic, irregular, or oval masses with microlobulated margins, while color Doppler may demonstrate internal hypervascularity. However, these imaging characteristics are nonspecific and can mimic other breast lesions, including malignancies. Histologically, AMEs are defined by a biphasic proliferation of epithelial and myoepithelial cells, with patterns such as spindle cell, tubular, and lobular variants. Immunohistochemical staining plays a crucial role in diagnosis, with markers such as p63, calponin, and smooth muscle myosin heavy chain being positive. Atypical features, including nuclear pleomorphism, mitotic activity, and necrosis, should prompt suspicion for malignancy. The primary treatment for AMEs is complete surgical excision with clear margins to reduce the risk of recurrence. Incomplete tumor removal is associated with a higher likelihood of local recurrence. Malignant transformation of either cellular component is rare but has been reported, with metastases to the lungs, brain, and thyroid. Malignant AMEs are characterized by significant cytologic atypia and high mitotic rates. Studies suggest that larger tumors with infiltrative margins carry an elevated risk of recurrence and metastasis. In this case report, we present a case of a 42-year-old female who presented for a routine screening mammography with a subsequent breast ultrasound and was found to have a suspicious mass that was pathologically proven as benign AME.

## Introduction

Adenomyoepitheliomas (AMEs) of the breast are rare neoplasms characterized by a biphasic proliferation of epithelial and myoepithelial cells. These tumors are predominantly present in middle-aged females between the ages of 40 and 70 years old [1-4]. Due to their rarity, limited cases are documented in the literature, and hence, these tumors present a unique diagnostic and clinical challenge [1,3-13].

Radiologically, AMEs often appear as irregular masses with suspicious features on mammography and ultrasound, necessitating further evaluation [1,3,5]. However, imaging characteristics are not pathognomonic, and definitive diagnosis relies on histopathological examination [1,5,8,11,12]. While the majority of AMEs are benign, there exists a potential for malignant transformation, underscoring the importance of accurate diagnosis and appropriate management [1-3,5-8,11-13]. Surgical excision with clear margins is the standard treatment to mitigate the risk of recurrence or progression [1-3,8,12].

In this report, we present a case of a benign AME of the breast, detailing its clinical presentation, imaging findings, histopathological features, and management. This case aims to contribute to the limited body of knowledge on AMEs and highlight the critical role of imaging in their diagnosis and treatment planning.

## Case presentation

**Figure 1:**
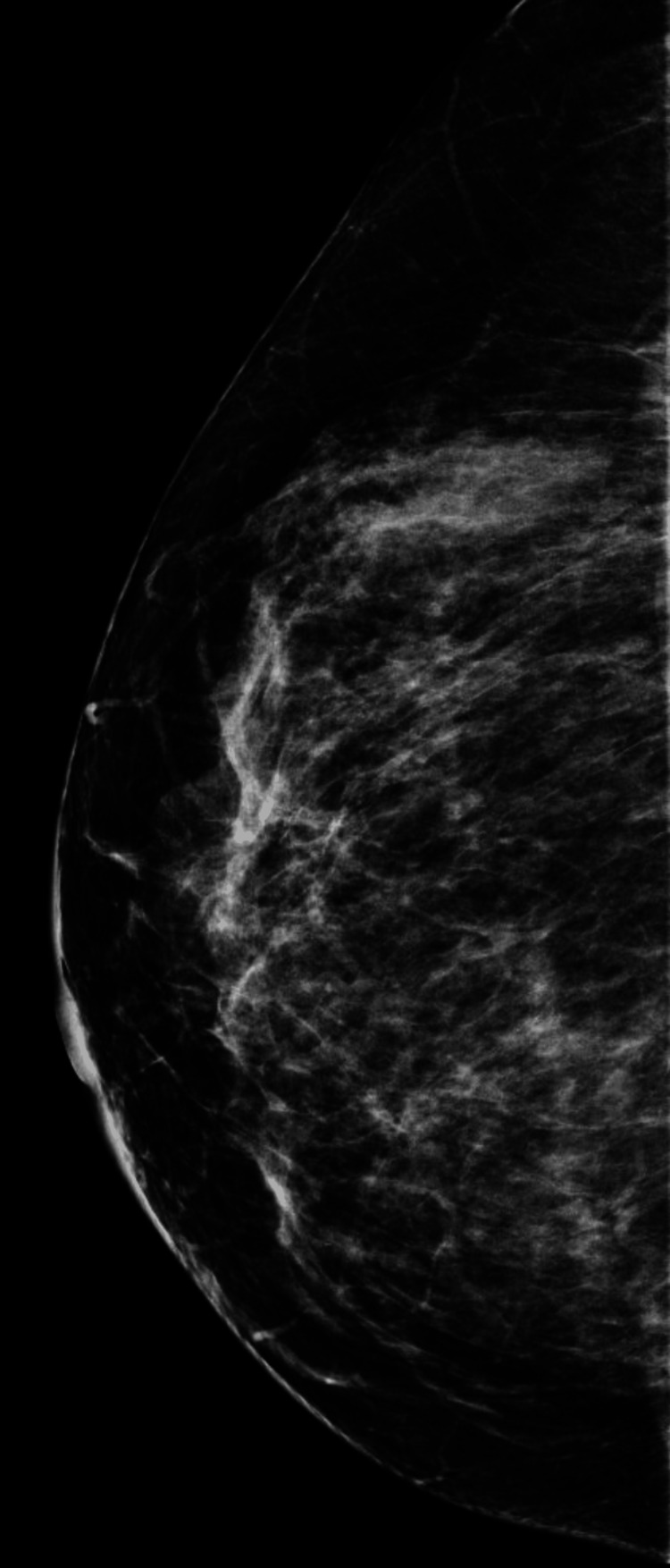
Craniocaudal view of the right breast on screening mammogram.

**Figure 2:**
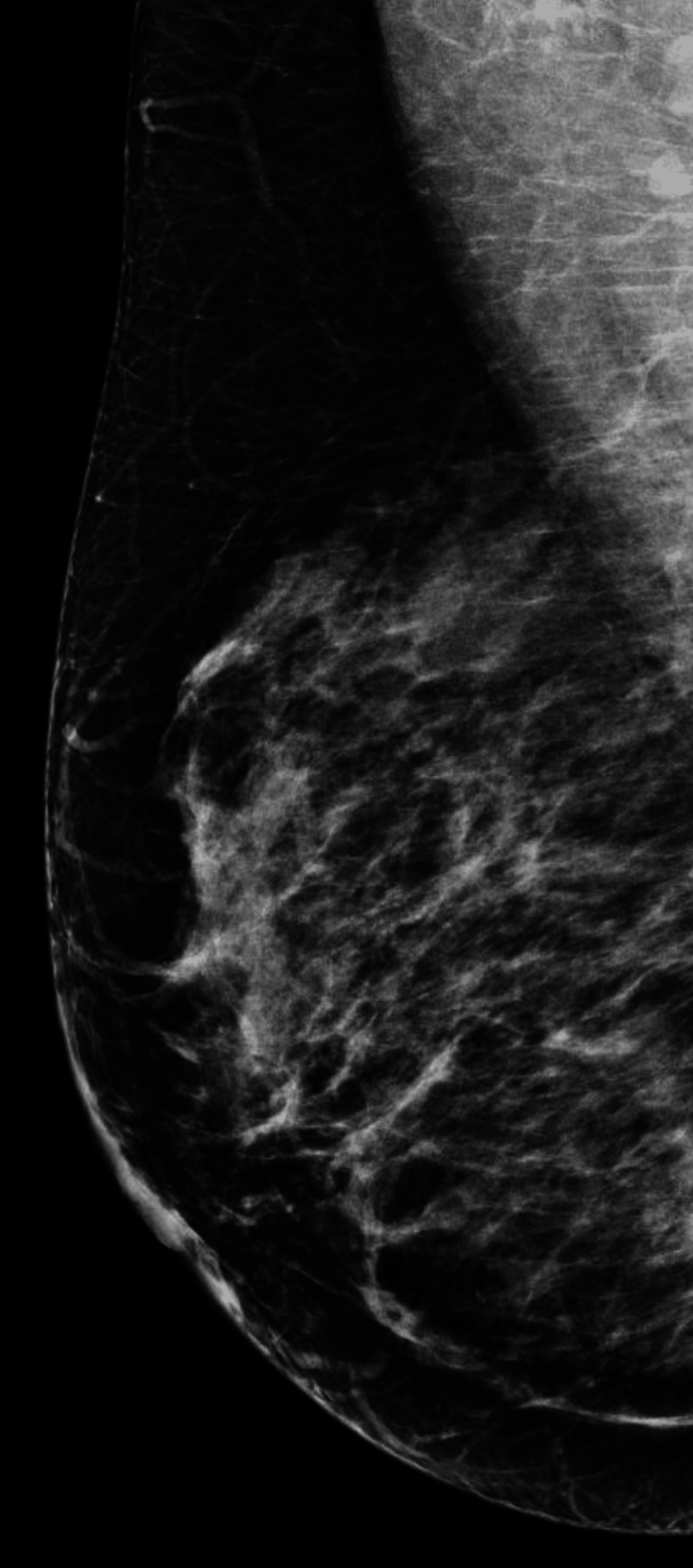
Mediolateral oblique view of the right breast on screening mammogram.

**Figure 3:**
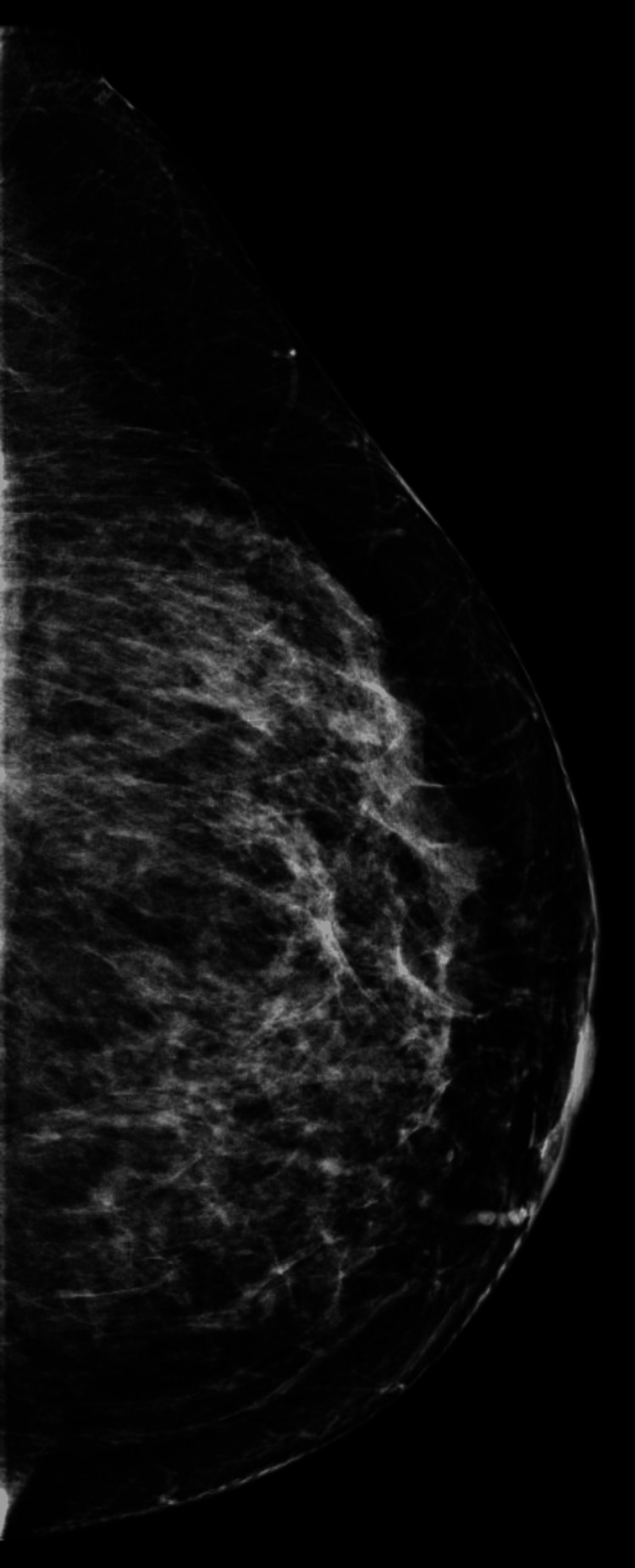
Craniocaudal view of the left breast on screening mammogram.

**Figure 4:**
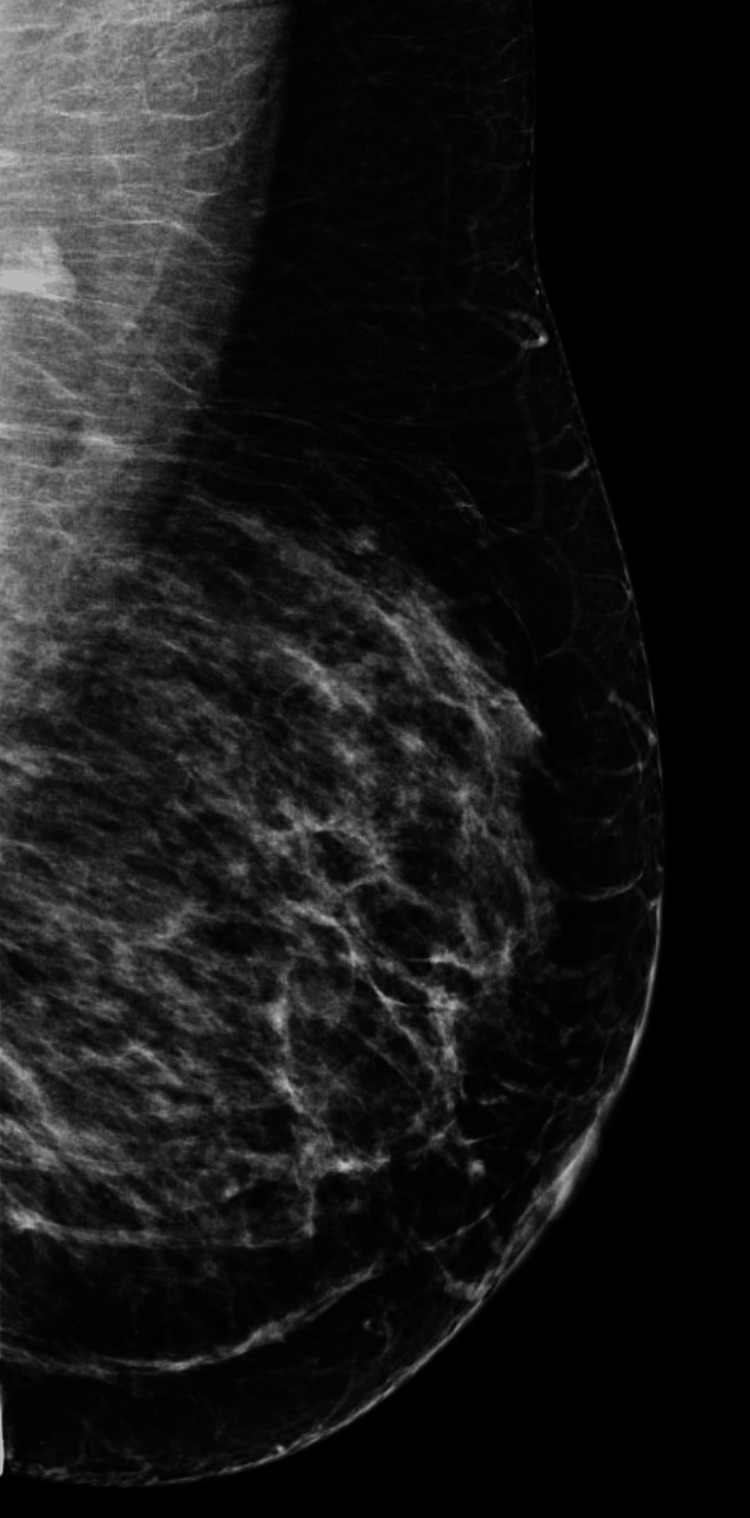
Mediolateral oblique view of the left breast on screening mammogram.

**Figure 5:**
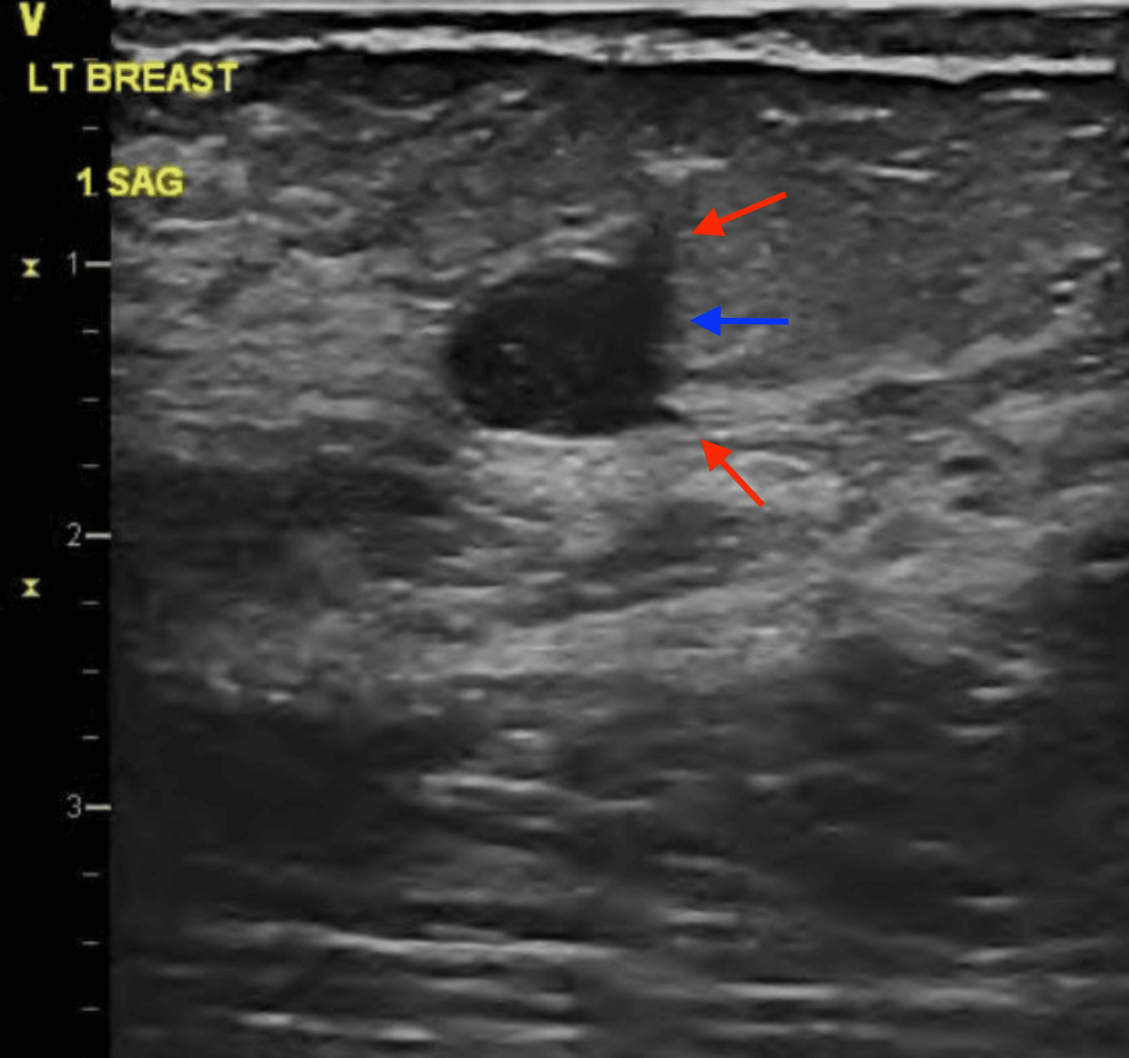
Ultrasound image showing a 0.76 x 0.92 cm oval, parallel, hypoechoic mass with indistinct and angulated margins in the left breast at the 1 o’clock position. Red arrows demonstrate angulated borders. The blue arrow demonstrates an indistinct margin.

**Figure 6:**
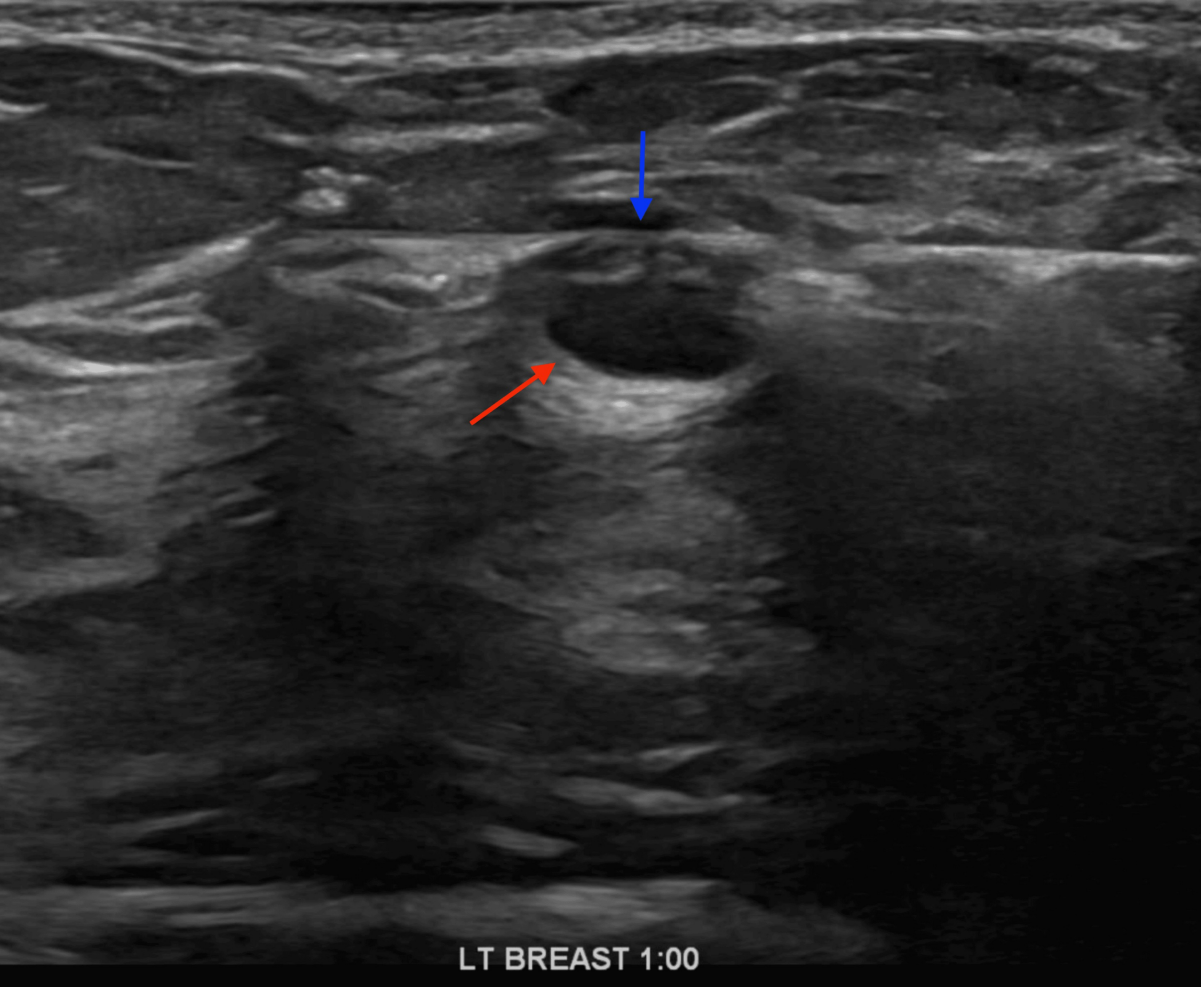
Left breast at the 1 o’clock position, showing a 14-gauge Celero biopsy core needle (Hologic, Inc., Marlborough, USA) targeting the mass under ultrasound guidance. The blue arrow demonstrates the core needle passing through the mass. The red arrow demonstrates the inferior border of the targeted mass.

**Figure 7:**
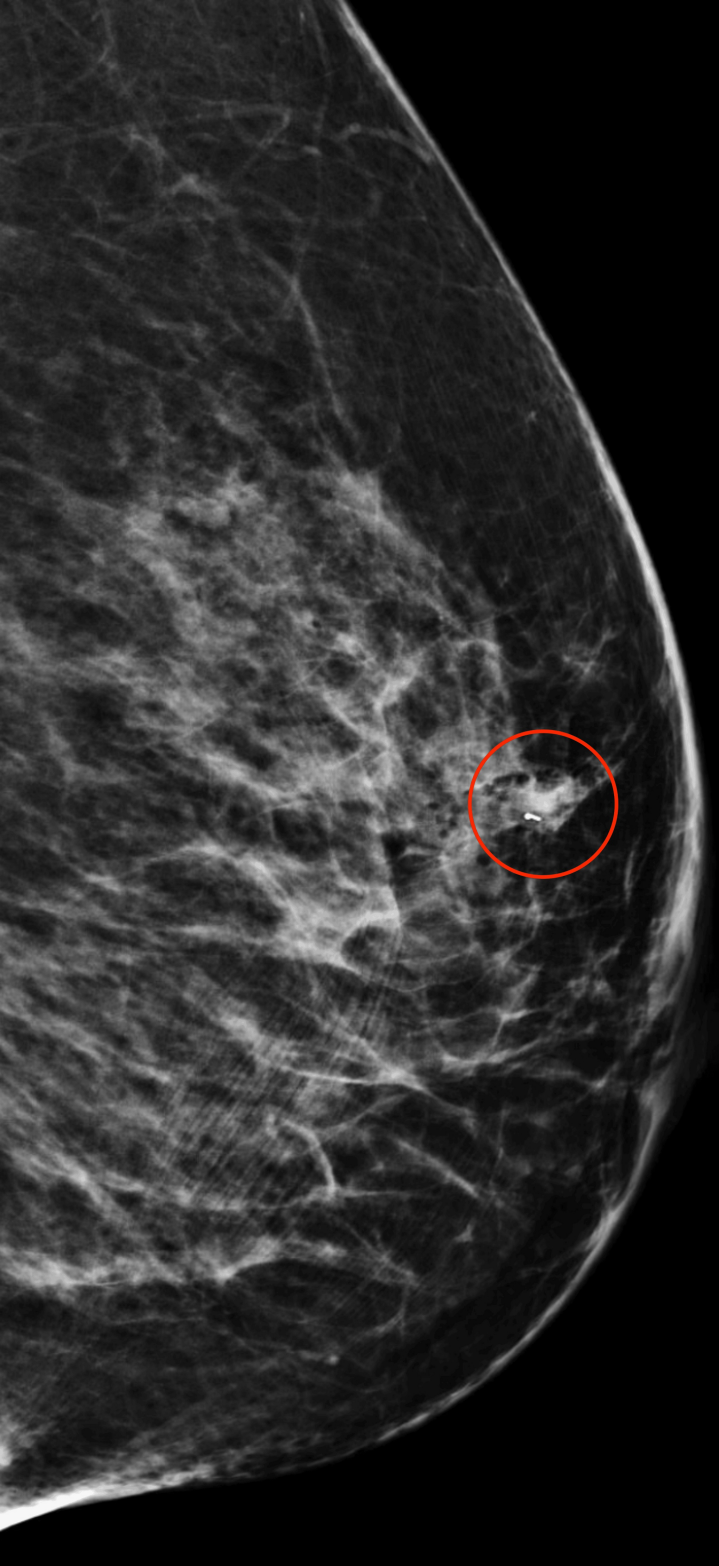
Left breast post-biopsy mammogram in mediolateral view. A red circle encircles a biopsy clip marker within an irregular, obscured mass of equal density in the left upper breast, approximately 3.5 cm from the nipple.

**Figure 8:**
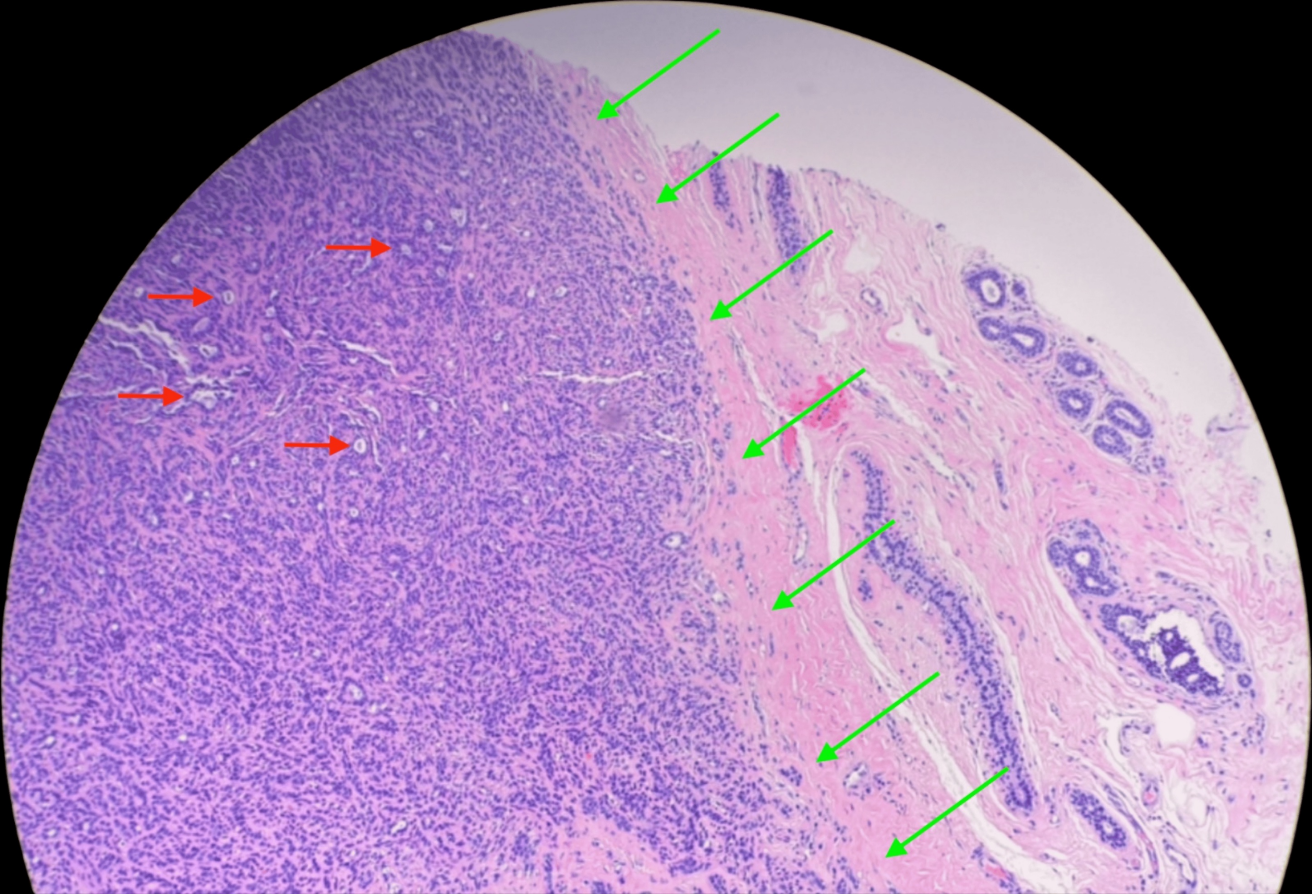
Low magnification hematoxylin and eosin (H&E) stain showing the mass composed of epithelial cells forming small glands and nests. Red arrows indicate small glands and nests. Green arrows outline the lesion.

**Figure 9:**
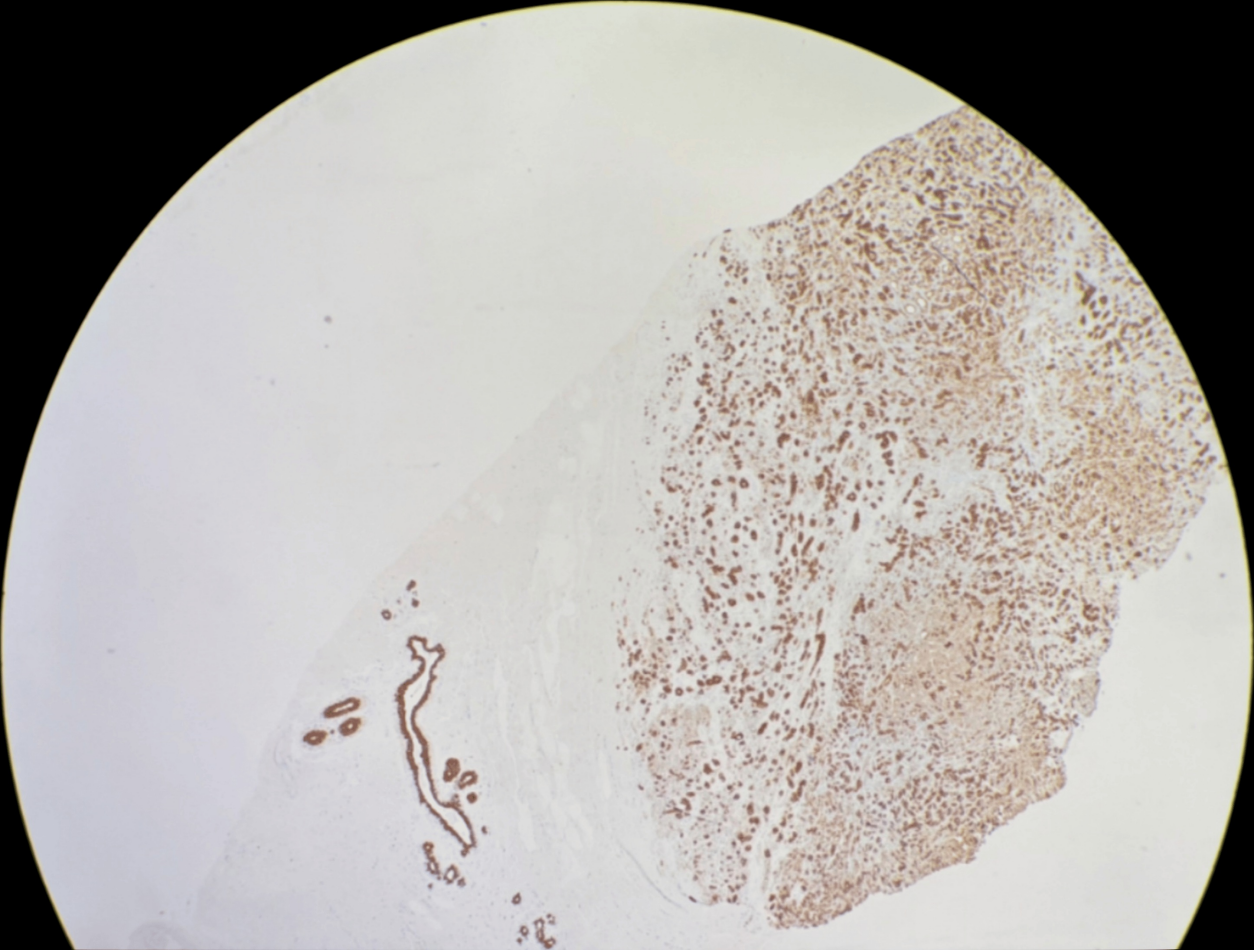
Low magnification pankeratin immunohistochemical stain showing diffuse positivity.

**Figure 10:**
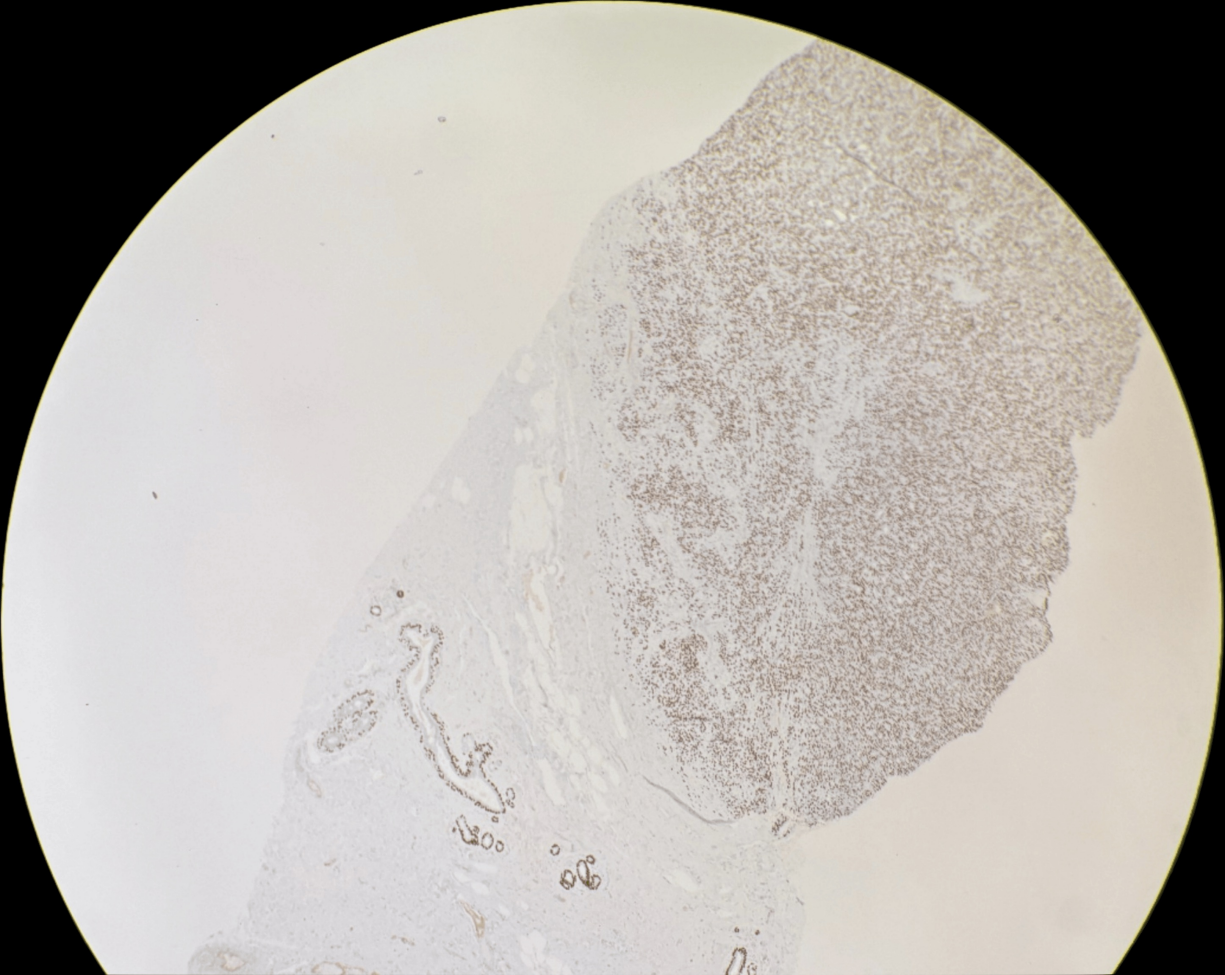
Low magnification p63 immunohistochemical stain showing diffuse positivity.

We present a 42-year-old asymptomatic Hispanic female with no personal or family history of breast cancer, who presented to our institution as an outside procedural referral for a left breast ultrasound-guided core needle biopsy after a suspicious mass was found on an outside diagnostic ultrasound. The patient underwent a screening mammogram in an outside facility, which demonstrated bilateral heterogeneously dense breasts (Figures 1-4). Dense breast tissue can mask or hide small cancers, making them more difficult to detect on mammograms. The patient returned approximately three weeks after to the outside facility where the breast ultrasound was performed. Findings on the ultrasound demonstrated a suspicious 0.92 cm mass in the left breast at the 1 o’clock position, approximately 3 cm from the nipple. The mass showed irregular, indistinct, hypoechogenicity and angulated margins, features suspicious for malignancy (Figure 5). A final diagnosis of Breast Imaging Reporting and Data System (BI-RADS) category 4 was given, and recommendations were made to undergo tissue sampling. The patient was then referred to our institution for an ultrasound-guided core needle. On the day of the procedure, an additional targeted left breast ultrasound of the 1 o’clock position redemonstrated a 0.92 cm mass with solid internal components and angulated margins, keeping with previously visualized malignant characteristics and confirming target area of concern. A biopsy was performed, acquiring three samples using a 14-gauge Celero biopsy core needle (Hologic, Inc., Marlborough, USA) under ultrasound guidance (Figure 6). A post-biopsy left breast mammogram confirmed clip marker placement in the mass (Figure 7). Tissue cores were sent to pathology later yielding AME with intraluminal calcifications and papilloma. Immunohistochemical stains performed showed myoepithelial cells positive for p63 and epithelial cells and positive for pankeratin and failed to demonstrate infiltrating epithelial cells (Figures 8-10). The patient was given a BI-RADS 4 and recommended for surgical consultation and excision. The patient opted against surgical intervention.

## Discussion

AME of the breast is a rare biphasic tumor characterized by the proliferation of epithelial and myoepithelial cells that encompass a wide spectrum of clinical presentations, imaging features, and histopathological patterns, contributing to diagnostic challenges [3-7,11,13]. This case highlights the critical role of integrating clinical, radiologic, and pathologic findings in the evaluation and management of AMEs [1-5,8,11,12].

Clinically, AMEs often present as painless, palpable masses, most frequently in middle-aged to elderly women [4,6,7,11,13]. Radiological features are nonspecific, with mammography and ultrasound findings often mimicking other benign or malignant breast lesions [3,5,8,11]. In this case, imaging suggested a suspicious mass, warranting biopsy for definitive characterization [7,8,11,13]. Such findings align with prior reports emphasizing the limited specificity of imaging in differentiating AMEs from other breast pathologies [3,5,8,11,13].

Histopathologically, AMEs are defined by the dual presence of epithelial and myoepithelial cell populations [3,6,8,10,13]. Immunohistochemical staining showed identification of myoepithelial markers (p63, smooth myosin) and epithelial markers (E-cadherin, pankeratin) [1,3,6,8,13]. In this case, there was an additional lesion of an intraductal papilloma in the core biopsy sample [2,9,11,12]. Although known, the co-existence of these two lesions further adds to the diagnostic challenge of AMEs, as partial sampling can lead to difficulty in distinguishing AMEs from papillomas, especially when the myoepithelial component is not well-represented [2,3,9,11]. AMEs are characterized by a biphasic proliferation of epithelial and myoepithelial cells, with the myoepithelial component often prominent and forming nests or nodules [1,7,10-12]. By contrast, papillomas are composed of a single layer of epithelial cells supported by fibrovascular cores and a basal myoepithelial layer, with less prominent myoepithelial proliferation [2,11,12]. Additionally, both entities contain epithelial and myoepithelial cells, making them histologically similar in some cases [1,4,7,11,13]. AMEs can also adopt a papillary growth pattern, which may mimic papillomas histologically [1,2,10,11].

Although the recommendations and literature favor undergoing surgical excision for AMEs as well as common practice for intraductal papillomas, this patient opted against any surgical management due to the benign nature of both masses [2,3,5,6,11]. Although benign, it is important to have a unified opinion on the management of these lesions as the majority of patients undergo multiple appointments with different medical specialties who may not necessarily know adequate recommendations and, although small, the risks of malignant transformation [1,2,5]. It is crucial to establish a unified approach to managing these lesions, as patients often require multiple consultations with various medical specialties that may lack familiarity with appropriate recommendations. Currently, there is no single, universally agreed-upon post-excision follow-up schedule for AMEs, and hence, continued yearly surveillance with diagnostic ultrasounds in adjunct with yearly mammograms seems a reasonable choice for follow-up.

## Conclusions

This case of AME highlights the diagnostic challenges posed by rare breast neoplasms. The integration of clinical, radiological, and histopathological findings is crucial in reaching an accurate diagnosis. While surgical excision is typically recommended for AMEs and intraductal papillomas, this case demonstrates the importance of patient autonomy in decision-making. The co-existence of AME with a papilloma in the biopsy sample further emphasizes the complexity of these lesions and the potential for misdiagnosis. This report underscores the need for a unified approach to managing benign breast lesions with malignant potential, particularly when multiple medical specialties are involved in patient care. By contributing to the limited literature on AMEs, we aim to enhance diagnostic accuracy and improve management strategies for these uncommon neoplasms.
